# Association between maternal food literacy and constipation prevalence in preschool children: a cross-sectional study

**DOI:** 10.1186/s40795-026-01384-3

**Published:** 2026-06-02

**Authors:** Akane Kojima, Kanae Wada, Yuka Tanaka, Kuniyasu Kamiya, Suzuka Gokyu, Masahiko Kato

**Affiliations:** 1 Department of Nutrition, Faculty of Health and Nutrition, Yamanashi Gakuin University, Kofu, Japan; 2 Child Care and Education, Yamanashi Gakuin Junior College, Kofu, Japan; 3 Basic Medical Sciences Region, Kobe City College of Nursing, Kobe, Japan; 4 Department of Human Nutrition, School of Life Studies, Sugiyama Jogakuen University, 17-3 Hoshigaokamotomachi Chikusa-ku, Nagoya, 464-8662 Japan

**Keywords:** Maternal food literacy, Childhood constipation, Cross-sectional study

## Abstract

**Background:**

To investigate the association between maternal food literacy and constipation prevalence in preschool-aged children.

**Methods:**

This cross-sectional study included 376 parents whose children attended kindergartens, child welfare facilities, and nursery schools. Maternal food literacy was identified using the validated self-perceived food literacy scale and children’s constipation prevalence using the Bristol Stool Form Scale. Logistic regression analysis was used to estimate odds ratios (ORs) and 95% confidence intervals (CIs).

**Results:**

Low maternal food literacy was associated with high constipation prevalence in children. After adjusting for child age, sex, and pharmacological treatments for constipation, multivariate-adjusted ORs for mothers of constipated children compared with those of non-constipated children were: healthy snack styles for per 1-point score decrease from (OR = 1.61, 95% CI: 1.05–2.48), daily food planning (OR = 1.40, 95% CI: 1.05–1.87), and healthy food stockpiling (OR = 1.56, 95% CI: 1.09–2.22). Lower total food literacy scores were associated with mothers of children with constipation (OR = 2.33, 95% CI: 1.15–4.72) compared to those without.

**Conclusions:**

These findings suggest an association between low maternal food literacy and a high prevalence of constipation in preschool children.

**Supplementary Information:**

The online version contains supplementary material available at 10.1186/s40795-026-01384-3.

## Background

Constipation is one of the most common reasons for pediatric medical visits and contributes substantially to increased healthcare costs [1]. A meta-analysis of Asian children aged 1–9 years reported a prevalence of 13.4% [2]. Childhood constipation, therefore, poses significant public health challenges and increases healthcare costs.

Diet plays an important role in the management of constipation [3]. Previous studies have reported an inverse association between constipation and the intake of specific foods and nutrients such as fruits, vegetables [4–6] and dietary fiber [6] in Asian children. However, the food industry is complex and constantly evolving. Therefore, understanding the impact of food-related behavior more comprehensively is crucial, not only in terms of the consumption of specific foods or nutrients, but also in terms of enabling individuals to choose healthier foods and eating habits [7]. This highlights the importance of understanding food-related literacy [7].

Food literacy is most commonly referenced as a set of interrelated knowledge, skills, and behaviors necessary to plan, manage, select, prepare, and consume food to meet needs and determine intake [7–9]. Younger children have less autonomy in managing their diet and nutrition than older children; therefore, they rely primarily on their mothers for food and nutrition [10]. Therefore, it is important to examine the relationship between maternal food literacy and the prevalence of constipation in children. However, to our knowledge, no previous study has investigated an association between maternal food literacy and the prevalence of constipation in children.

To provide information on the theoretical basis and practical strategies for preventing constipation in children, it is necessary to investigate the relationship between maternal food literacy and childhood prevalence of constipation. This cross-sectional study examined the association between food literacy of mothers and the prevalence of constipation in preschool-aged children.

## Materials and methods

### Study population

**Fig. 1:**
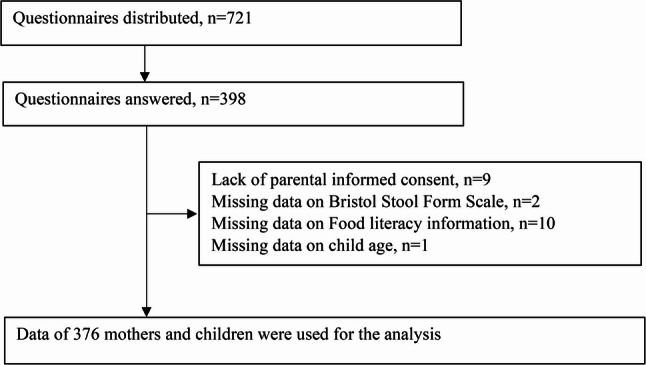
Participant selection flow chart

This study used cross-sectional data from parents and their children enrolled in kindergartens, child welfare facilities, and nursery schools between August and September 2025. Data on food literacy, constipation, and other explanatory variables were collected using self-reported online questionnaires completed by parents who consented to participate in this study (response rate: 55.2%). The questionnaire used was created for this study (see Supplementary Material 1). Among the 398 parents of healthy children initially recruited, we excluded individuals with missing informed consent (*n* = 9), missing data on the Bristol Stool Form Scale (BSFS: *n* = 2), missing maternal food literacy information (*n* = 10), and missing data on children’s ages (*n* = 1), respectively. Ultimately, 376 parents of healthy children were included in the study (Fig. 1).

### Constipation

The parents rated their children’s stool consistency using the BSFS [11]. To assess the BSFS, the online questionnaire included detailed descriptions of seven stool types (although visual images were provided in the actual survey, they have been replaced with text descriptions in the Supplementary Material 1 for copyright compliance) (type 1: hard lumps, like nuts; type 2: sausage-like, but lumpy; type 3: like a sausage but with cracks on the surface; type 4: like a sausage or snake, smooth and soft; type 5: soft blobs with clear-cut edges; type 6: fluffy pieces with ragged edges, a mushy stool; and type 7: watery, no solid pieces). Following previous reports [12, 13], children’s stool types 1 and 2 were defined as having constipation.

### Food literacy

Maternal self-perceived food literacy (SPFL) was assessed using the Japanese version [9] of the previously reported SPFL scale. The Japanese version is a translation of the original English SPFL scale [9, 14] developed by the second author in a previous publication [9]. The back-translation was reviewed by the original developers [14], and revisions were made to ensure that the translated version accurately reflected the original scale items. The SPFL scale, consisting of 29 items, assesses eight different domains of knowledge about food, and its validity has been established in other studies [9, 14]. In Murakami et al.’s study [9], the cohort consisted of 1,055 Japanese adults with an average age of 44 years, of whom approximately 50% were women. Murakami et al.’s study reported that the Cronbach’s alpha—for evaluating the internal consistency of the Japanese version of the SPFL scale—in this sample was within the range of values considered satisfactory (0.58–0.97), with the exception of resilience and resistance (0.57) and social and conscious eating (0.51) [9]. Using the SPFL Scale—an established and reliable tool—, mothers were asked to answer all questions based on a 5-point Likert scale (1 = “never/never” to 5 = “yes/always”) [9]. The SPFL total score was calculated as the average of all items, and negatively worded items were reverse-coded; higher scores indicated greater food literacy [9, 15]. The scores for each domain were calculated by dividing the sum of the scores by the number of items [9, 15].

### Other explanatory variables

A structured, self-reported online questionnaire was used to obtain information on the following variables: (1) child factors: age (continuous variable, 3–6 years), sex (female or male), sleep duration (< 7, 8–9, 10–11, 12–13, and ≥ 14 h/day), daily screen time (continuous variable; [min]), constipation medication (yes or no), vegetable intake frequency (< 1 time/day or ≥ 1 time/day), fruit intake frequency (< 1 time/day or ≥ 1 time/day), and water and fluid intake (1 cup [200 mL/day], 2 cups [400 mL/day], 3 cups [600 mL/day], 4 cups [800 mL/day], 5 cups [1000 mL/day], ≥ 6 cups [1200 mL/day]). (2) Maternal factors: age (10–19, 20–29, 30–39, 40–49, 50–59, ≥ 60 years, and do not want to answer), education level (high school, vocational school, junior college, university and higher, prefer not to answer), employment status (full-time housewife, part-time/casual, regular employee, prefer not to answer), drinking status (never, past, and current), and smoking status (never, past, and current).

The variables were categorized as follows: child sleep duration (< 10 h/day vs. ≥10 h/day), child water and fluid intake (≤ 800 mL/day vs. >800 mL/day), maternal education level (less than university vs. university or higher), maternal employment status (non-regular employees vs. regular employees), maternal drinking status (never or past vs. current), maternal smoking status (never or past vs. current).

### Statistical analysis

Continuous variables are presented as mean (standard deviation [SD]), and between-group comparisons were performed using Student’s t-test. Categorical variables were presented as percentages (%), and chi-square tests were used for between-group comparisons.

Logistic regression analysis was used to analyze the association between maternal food literacy and constipation in children. These associations were adjusted for the child’s age and sex, as well as other variables that were significantly associated with the presence or absence of constipation in this study, and were estimated using the following model: (1) Multivariate model I, adjusted for child age, sex, and pharmacological treatments for constipation. (2) Multivariate model II, further adjusted for the frequency of vegetable intake in children. The Stata statistical software package (version 16; Stata Corp LLC, College Station, TX, USA) was used for all statistical analyses, with a significance level of *P* < 0.05.

## Results

Table 1 shows the characteristics of preschool children with and without constipation. The prevalence of constipation in this cohort of preschool children was 14.6%. Compared with non-constipated children, those with constipation had a higher rate of child pharmacological treatment for constipation (*P* = 0.01). Moreover, a higher proportion of preschool children with constipation consumed vegetables < 1 time/day (*P* = 0.04) and sugar-sweetened beverages (SSB) ≥ 1 time/day (*P* = 0.09) compared with those without constipation.

**Table 1:** Characteristics of Preschool Children With and Without Constipation

	All	Constipation		*P* value
		Without	With	
Participants, *n*	376	321	55	ー
Age, years (mean)	4.42 ± 0.97	4.45 ± 0.98	4.27 ± 0.93	0.22
Sex, male (%)	52.7	53.3	49.1	0.57
Sleep duration, < 10 h/day (%)	55.6	55.1	58.2	0.68
Screen time, min/day (mean ± SD)	91.4 ± 58.8	91.7 ± 60.4	89.5 ± 48.9	0.80
Pharmacological treatment for constipation, yes (%)	4.3	3.1	11.1	0.01
Frequency of vegetables, < 1 time/day (%)	7.7	6.5	14.5	0.04
Frequency of fruit, < 1 time/day (%)	43.6	42.9	47.3	0.55
Frequency of sugar sweetened beverages, ≥ 1 time/day (%)	23.7	22.2	32.7	0.09
Frequency of sweets, ≥ 1 time/day (%)	71.7	71.2	74.5	0.61
Water and fluid intake, ≤ 800 mL/day (%)	61.1	59.7	69.1	0.19

Values indicate the mean ± SD or the proportion

Since participants with missing values for each variable were excluded, the available sample sizes for all participants (with and without constipation) were as follows: for screen time, 373; for pharmacological treatment for constipation, frequency of fruit, and frequency of sweets, 374; for frequency of sugar sweetened beverages and water and fluid intake, 375

Participant characteristics according to whether they were with or without constipation were evaluated using the chi-square test or unpaired t-test

Table 2 shows the characteristics of mothers of preschool children with and without constipation. Compared with mothers of children without constipation, those with constipation had lower maternal food literacy scores in the domains of healthy snack styles (*P* = 0.04), daily food planning (*P* = 0.02), healthy food stockpiling (*P* = 0.02), and total food literacy (*P* = 0.02).

**Table 2:** Characteristics of Mothers of Preschool Children With and Without Constipation

	All	Constipation		*P* value
		Without	With	
Participants, *n*	376	321	55	ー
Age, years (%)				
10–19	0.5	0.6	0.0	0.85
20–29	5.1	5.3	3.6	
30–39	62.5	61.4	69.1	
40–49	30.9	31.5	27.3	
50–59	0.3	0.3	0.0	
60 years and older	0.0	0.0	0.0	
Do not want to answer	0.8	0.9	0.0	
Education level, university and higher (%)	41.5	41.7	40.0	0.81
Employment status, regular employee (%)	44.4	44.9	41.8	0.68
Drinking, current (%)	36.5	35.9	40.0	0.56
Smoking, current (%)	5.6	5.6	5.5	0.96
Food literacy domains (mean ± SD)				
Food preparation skills	3.9 ± 0.7	3.9 ± 0.7	3.9 ± 0.7	0.58
Resilience and resistance	3.3 ± 0.6	3.3 ± 0.6	3.2 ± 0.6	0.56
Healthy snack styles	2.4 ± 0.7	2.4 ± 0.7	2.2 ± 0.7	0.04
Social and conscious eating	4.0 ± 0.6	4.1 ± 0.6	3.9 ± 0.6	0.17
Examining food labels	2.7 ± 1.1	2.7 ± 1.1	2.6 ± 1.2	0.59
Daily food planning	3.6 ± 0.9	3.6 ± 0.9	3.3 ± 1.1	0.02
Healthy budgeting	3.7 ± 0.9	3.7 ± 0.9	3.5 ± 0.8	0.20
Healthy food stockpiling	3.6 ± 0.8	3.7 ± 0.8	3.4 ± 0.9	0.02
Total food literacy (mean ± SD)	3.4 ± 0.4	3.4 ± 0.4	3.3 ± 0.4	0.02

Values indicate the mean ± SD or the proportion

Since participants with missing values for each variable were excluded, the available sample sizes for all participants (with and without constipation) were as follows: for drinking, 375; and for smoking, 374

Participant characteristics according to whether they were with or without constipation were evaluated using the chi-square test or unpaired t-test

Table 3 shows the crude and multivariate odds ratios (ORs), and 95% confidence intervals (CIs) for constipation among preschool children in terms of maternal food literacy. Low maternal food literacy was associated with a high prevalence of constipation in children. After adjusting for the child’s age, sex, and pharmacological treatment for constipation, the multivariate-adjusted ORs for each domain of food literacy in mothers of children with constipation, compared with those of children without constipation were as follows: healthy snack styles for per 1-point score decrease ranged from (OR = 1.61, 95% CI: 1.05–2.48), daily food planning (OR = 1.40, 95% CI: 1.05–1.87), and healthy food stockpiling (OR = 1.56, 95% CI: 1.09–2.22). Lower total food literacy scores for a 1-point score decrease, calculated as the average of all maternal food literacy items, were associated with increased constipation in children (OR = 2.33; 95% CI: 1.15–4.72) (multivariate model Ⅰ). Furthermore, the multivariate-adjusted ORs adjusted for children’s vegetable intake frequency were 1.55 (95% CI: 1.00–2.40) for healthy snack styles, 1.38 (95% CI: 1.03–1.85) for daily food planning, 1.51 (95% CI: 1.05–2.17) for healthy food stockpiling, and 2.05 (95% CI: 0.99–4.25) for total food literacy (multivariate model II).

**Table 3:** The Crude and the Multivariable ORs (95% CI) for Constipation among Preschool Children on the Maternal Food Literacy

	Crude model ^1^	*P* value	Multivariate model I ^2^	*P* value	Multivariate model Ⅱ ^3^	*P* value
Maternal food literacy domains (per 1-point decrease)						
Food preparation skills	1.12 (0.75–1.65)	0.58	1.06 (0.70–1.59)	0.78	0.96 (0.63–1.47)	0.86
Resilience and resistance	1.16 (0.71–1.87)	0.56	1.20 (0.73–1.97)	0.48	1.13 (0.69–1.88)	0.62
Healthy snack styles	1.57 (1.03–2.39)	0.04	1.61 (1.05–2.48)	0.03	1.55 (1.00–2.40)	0.05
Social and conscious eating	1.39 (0.87–2.21)	0.17	1.43 (0.89–2.30)	0.14	1.35 (0.83–2.20)	0.22
Examining food labels	1.07 (083–1.40)	0.59	1.11 (0.84–1.45)	0.47	1.08 (0.82–1.41)	0.59
Daily food planning	1.39 (1.05–1.85)	0.02	1.40 (1.05–1.87)	0.02	1.38 (1.03–1.85)	0.03
Healthy budgeting	1.23 (0.89–1.70)	0.21	1.25 (0.90–1.75)	0.18	1.20 (0.86–1.69)	0.29
Healthy food stockpiling	1.51 (1.07–2.13)	0.02	1.56 (1.09–2.22)	0.02	1.51 (1.05–2.17)	0.03
Maternal total food literacy (per 1-point decrease)	2.20 (1.11–4.36)	0.02	2.33 (1.15–4.72)	0.02	2.05 (0.99–4.25)	0.05

*OR*  odd ratio, * CI * confidence interval

^1^ The data of 376 children were used for this analysis; the total constipation count was 55

^2^ The data of 374 children were used for this analysis; the total constipation count was 54

^2^ Based on multivariate model I, the explanatory variables included sex (male or female), age (years, continuous), and prescription of child pharmacological treatment for constipation (yes or no)

^3^ The data of 374 children were used for this analysis; the total constipation count was 54

^3^ Based on multivariate model Ⅱ, the explanatory variables included the listed variables, sex (male or female), age (years, continuous), child pharmacological treatment for constipation (yes or no), and frequency of vegetables (< 1 time/day or ≥ 1 time/day)

## Discussion

This cross-sectional study demonstrated an association between lower maternal food literacy and higher prevalence of constipation in preschool children. After adjusting for child age, sex, and pharmacological treatments for constipation, lower scores for healthy maternal snack style, daily food planning, healthy food stockpiling, and total food literacy were associated with an increased prevalence of constipation in children.

In our study, a positive association was found between an increase in the prevalence of constipation and lower scores in the food literacy domains of maternal healthy snack styles (always having vegetables and fruits ready when going out or as snacks and consuming them) and healthy food stockpiling (stockpiling snack foods, sweets, and SSBs). The Dietary Guidelines for Americans 2015–2020 recommends the consumption of good sources of dietary fiber, such as vegetables and fruits for constipation in children [16]. In contrast, a higher intake of ultra-processed foods has been reported to be associated with a higher intake of added sugars and saturated fats, and epidemiological studies have confirmed that sugary products are associated with an increased risk of constipation, as they have been shown to prolong intestinal transit time [17]. Furthermore, it has been suggested that SSBs may interfere with peristalsis and intestinal barrier function, allowing harmful substances to penetrate the intestine and increasing the risk of constipation [12]. A cross-sectional study of 119 US children [18] reported that the consumption of vegetables and fruits on the go or as snacks accounted for 16.9% of the daily vegetable and fruit intake. Similarly, a cross-sectional study of 111 Norwegian preschool parents and children [19] reported that mothers’ who created healthier food environments had children with higher intake of fruits (OR = 1.99, 95% CI = 1.15) and berries (OR = 2.1, 95% CI = 1.17, 3.78), vegetables (OR = 2.94, 95% CI = 1.55, 5.55), and lower intake of high-sugar foods (OR = 0.54, 95% CI = 0.29, 0.98). Our study found that the association between the mothers’ healthy snack styles and their children’s constipation weakened after adjusting for the frequency of their children’s vegetable intake. Therefore, lower maternal food literacy regarding healthy snack styles may be associated with an increased prevalence of constipation in children, mediated by a lower frequency of vegetable intake in their children. Similarly, the results of this study suggest that the consumption of SSBs ≥ 1 time/day tended to be higher in constipated children than in non-constipated children. Thus, lower maternal healthy food stockpiling may also play a role in determining the exposure to and availability of healthy foods, which may be associated with an increased prevalence of constipation in children. 

Low maternal food literacy in the daily food planning domain may be associated with an increased prevalence of constipation in children, partly due to their inability to provide healthy meals. This finding is partially supported by previous studies. A longitudinal study of 286 Chinese undergraduate students [20] found that a high intention to eat at least five servings of fruit and vegetables per day was a predictor of higher subsequent intake. A cross-sectional study of 40,554 French adults [21] reported that meal planning, that is, planning food consumption for the next few days, was associated with increased adherence to nutritional guidelines. A cross-sectional study of 13,945 US adults [22] reported that higher adherence to the Healthy Eating Index (HEI)-2015 may be associated with a lower prevalence of constipation.

Finally, this study found an association between a decrease in maternal total food literacy score and an increase in prevalence of constipation in children. The development of children’s eating habits is complex and strongly influenced by maternal beliefs and attitudes toward food, as well as their emotional and cultural relationships with food [23]. Additionally, mothers play an important role, as the maternal child-rearing environment is one of the first and most fundamental contexts in which children acquire healthy food literacy and develop desirable eating habits [23]. Therefore, assessing maternal food literacy in preventing constipation in preschool children as a total domain may be more informative than when assessed as an individual domain.

To our knowledge, this is the first study to investigate the association between maternal food literacy and the prevalence of constipation in children. However, this study had some limitations. First, since this study had a cross-sectional design, a causal relationship between maternal food literacy and childhood constipation could not be established. Second, although we adjusted for children’s ages, sex, pharmacological treatment for constipation, and frequency of vegetables, we cannot rule out the possibility of unmeasured residual confounding factors. For example, family relationships, particularly parenting styles [24], were not assessed. Third, the definition of constipation in this study was primarily based on the Bristol Stool Form Scale (BSFS). Although the BSFS is a well-validated and objective tool for assessing stool consistency. Variables such as bowel movement frequency, parental concerns regarding painful bowel movements, and stool withholding behavior [25, 26] were not included. Therefore, future studies should assess the prevalence of constipation in combination with BSFS and these variables.

## Conclusions

This study suggests an association between low maternal food literacy and a high prevalence of constipation in preschool children.

## Supplementary Information

Supplementary Material 1.

## Data Availability

The data that support the findings of this study are not publicly available for privacy reasons but are available from the corresponding author upon reasonable request.

## Declarations

This study included parents and children, and informed consent was obtained from all parents through the website.

## Abbreviations

BSFS: Bristol Stool Form Scale
CI: Confidence intervals
HEI: Healthy Eating Index
OR: Odds Ratios
SD: Standard deviation
SPFL: Self-perceived food literacy
SSB: Sugar-sweetened beverages
