# Supplementary material for: Association between maternal food literacy and constipation prevalence in preschool children: a cross-sectional study
Source: BMC Nutr. 2026 Jun 2;12:159. doi: 10.1186/s40795-026-01384-3 (PMC13465892; doi:10.1186/s40795-026-01384-3)
Supplement: Supplementary file 1 [file 40795_2026_1384_MOESM1_ESM.docx]

**Research on children's lifestyles and health**

**This survey is aimed at children and their parents.**

**There are 78 questions in total (it will take approximately 15 minutes to complete).**

1. I fully understand the purpose of the research on children's lifestyle habits and health, and the contents of the explanatory document (Document 2) that describes the protection of personal information. Therefore, I agree to participate in this study (by answering this questionnaire). If you agree, please check "I agree" below and then begin answering the questionnaire. If you withdraw your consent after submitting the form, the data you provide will be discarded.

□　I agree

2. Please enter the date of consent here.

　　　　　　　　　　　　　　　　　　 Example: 20241108

３. Please enter the name of the parent

who has consented to participate.

*If your name is not entered, we may not be able to distribute the Thank You book card.

　　　　　　　　　　　　　　　 Example: Hanako Yamanashi

４．Please enter your child's childcare facility.

　　　　　　　　　　　　　　　　 Example: ●● Nursery School or ●● Child Welfare Facility

5．Please enter your child's class name.

　　　　　　　　　　　　　　　　 Example: Blue Class

**Questions regarding your child.**

6. Please enter your child's current height (cm).

　　　　　　　　　 Example: 125.4

7. Please enter your child's current weight (kg).

　　　　　　　　　 Example: 16.4

8. Please select the gender of your child.

| 1. Male | 1. Female |
| --- | --- |

9. Please select the birth order of your child.

| 1. 3 years old | 1. ４ years old | 1. ５ years old | 1. ６ years old |
| --- | --- | --- | --- |

10. Please select the birth order of your child.

| ① First child | ② Second child | ③ Third child | ④ More |
| --- | --- | --- | --- |

**Questions regarding your child's eating habits over the past three months.**

11. How often does your child consume grains (rice, wheat, buckwheat, mochi, bread, udon, spaghetti, corn flakes, etc.)?

| ① Less than once a day | ② Once or more daily |
| --- | --- |

12. How often does your child consume seafood (raw fish, salted fish, dried fish, shellfish, shrimp, squid, octopus, canned tuna, chikuwa, hanpen, kamaboko, etc.)?

| ① Less than once a day | ② Once or more daily |
| --- | --- |

13. How often does your child consumes meat (beef, pork, chicken, ham, sausage, etc.)?

| ① Less than once a day | ② Once or more daily |
| --- | --- |

14. How often does your child consume eggs?

| ① Less than once a day | ② Once or more daily |
| --- | --- |

15. How often does your child consume soybeans and soybean products (soybeans, tofu, thick deep-fried tofu, thin deep-fried tofu, okara, frozen tofu, yuba, natto, etc.)?

| ① Less than once a day | ② Once or more daily |
| --- | --- |

16. How often does your child eat vegetables?

| ① Less than once a day | ② Once or more daily |
| --- | --- |

17. How often does your child eat fruit?

| ① Less than once a day | ② Once or more daily |
| --- | --- |

18. How often does your child consume milk and dairy products (milk, yogurt, cheese, etc.)?

| ① Less than once a day | ② Once or more daily |
| --- | --- |

19. How often does your child consume unsweetened beverages, such as tea?

| ① Less than once a day | ② Once or more daily |
| --- | --- |

20. How often does your child consume sweetened beverages?

| ① Less than once a day | ② Once or more daily |
| --- | --- |

21. How often does your child eat sweets?

| ① Less than once a day | ② Once or more daily |
| --- | --- |

22. How often does your child eat instant ramen or cup ramen?

| ① Less than once a day | ② Once or more daily |
| --- | --- |

23. How often does your child eat fast food?

| ① Less than once a day | ② Once or more daily |
| --- | --- |

24. How often does your child eat fermented foods (natto, yogurt, cheese, rice bran pickling, pickles, soy sauce, miso, vinegar, mirin, shio-koji, amazake, etc.)?

| ① Less than once a month | ② 1-3 times a month | ③ 1-3 times a week | ④ 4-6 times a week | ⑤ Once a day | ⑥ 2-3 times a day |
| --- | --- | --- | --- | --- | --- |
| ⑦ 4 or more times a day |  |  |  |  |  |

25. How much fluid does your child consume per day (including tea and juice)?

| ① Less than 1 cup (200ml) | ② 1 cup (200ml) | ③ 2 cups (400ml) | ④ 3 cups (600ml) | ⑤ 4 cups  (800ml) |
| --- | --- | --- | --- | --- |
| ⑥ 5 cups (1000ml) | ⑦ 6 cups (1200ml) or more |  |  |  |

**Questions regarding your child's sleep duration.**

26. On average, how many hours of sleep does your child get on a weekday?

| ① Less than 7 hours | ② 8-9 hours | ③10-11 hours | ④12-13 hours | ⑤ 14 hours or more |
| --- | --- | --- | --- | --- |

**Questions regarding your child’s physical activities.**

27. On average, how many days per week does your child engage in moderate physical activity (such as brisk walking, swimming [slow backstroke or breaststroke], cycling, radio calisthenics, baseball, ballet, etc.)? Please do not consider walking for this answer.

| ① None | ② One day | ③ Two days | ④ Three days | ⑤ Four days |
| --- | --- | --- | --- | --- |
| ⑥ Five days | ⑦ Six days | ⑧ Seven days |  |  |

28. How many minutes of moderate physical activity does your child do per day?

　　　　　　　 Example: 65

29. On average for week, how many minutes per day does your child spend watching TV, playing digital games (including mobile phone games), or watching YouTube? *If none, enter 0.

　　　　　　　 Example: 120

**Questions regarding your child.**

30. Is your child currently taking any of the following medications, prescribed by a doctor (Multiple

choices allowed)?

| ① None | ② Constipation medication | ③ Allergy medication |
| --- | --- | --- |

31. How was your child fed during the first month after birth?

| ① Breast milk only | ② Breast milk and formula | ③ Formula only |
| --- | --- | --- |

**Questions regarding your child's cavities.**

32. Please tell us about any cavities you have had up to the present time (at the time of answering the questionnaire).

| ① No cavities | ② Teeth with completed cavity treatment | ③ Teeth with completed cavities and teeth with untreated cavities | ④ Untreated cavities |
| --- | --- | --- | --- |

33. Daily toothbrushing frequency

| ① ０ times | ② 1 time | ③ More than twice |
| --- | --- | --- |

34. Do your child's undergo regular dental checkups?

| ① Yes | ② No |
| --- | --- |

**Please tell us about your bowel movements.**

35. Has your child been diagnosed with constipation by a doctor?

| ① Yes | ② No |
| --- | --- |

36. Has your child been diagnosed with irritable bowel syndrome by a doctor?

| ① Yes | ② No |
| --- | --- |

37. Does your child have two or fewer bowel movements per week?

| ① Two times or fewer | ② Three or more times |
| --- | --- |

38. Which type best describes your child’s usual stool? Please choose the most appropriate option.

| Type 1 | Type 2 | Type 3 | Type 4 | Type 5 | Type 6 | Type 7 |
| --- | --- | --- | --- | --- | --- | --- |
| Separate hard lumps, like nuts (hard to pass) | Sausage-like, but lumpy | Like a sausage but with cracks in the surface | Like a sausage or snake, smooth and soft | Soft blobs with clear-cut edges | Fluffy pieces with ragged edges, a mushy stool | Watery, no solid pieces |

(Note: The text descriptions of stool form are based on Bristol Stool Form Scale)

**Questions regarding your child's allergies.**

39. Has your child been diagnosed with any of the following allergies by a doctor?

Food allergy, atopic dermatitis, allergic rhinitis, hay fever, allergic conjunctivitis, bronchial asthma *(asthma)*

| ① Yes | ② No |
| --- | --- |

40. Please select all that apply for the allergies that your child has been diagnosed with by a doctor (Multiple choices allowed).

| ① Food allergies | ② atopic dermatitis | ③ allergic rhinitis |
| --- | --- | --- |
| ④ hay fever | ⑤ allergic conjunctivitis | ⑥ bronchial asthma (*asthma*) |

**Questions regarding you or the child’s other parent’s allergies.**

41. Has your child's mother and/or father been diagnosed with any of the following allergies?

Food allergies, atopic dermatitis, allergic rhinitis, hay fever, allergic conjunctivitis, and/or bronchial asthma (*asthma*)

| ① Yes | ② No |
| --- | --- |

42. Which allergies has your child's mother and/or father been diagnosed with?

Please select those that apply. (Multiple answers are possible)

| ① Food allergies | ② atopic dermatitis | ③ allergic rhinitis |
| --- | --- | --- |
| ④ hay fever | ⑤ allergic conjunctivitis | ⑥ bronchial asthma (*asthma*) |

**Please tell us about the following: even if the questions seem similar, please answer all of them. Choose the answer that best applies to you (the child's mother). There are no wrong answers. All answers are correct.**

| The questions below are about how you select and handle ingredients, and about your cooking skills. | ― | ― | ― | ― | ― |
| --- | --- | --- | --- | --- | --- |
| Q43 When you eat something, do you take account of what you will eat later that day? | Not at all | Hardly ever | It depends | Mostly | Always |
| Q44 When you eat something, do you reflect on what you have eaten earlier that day? | Not at all | Hardly ever | It depends | Mostly | Always |
| Q45 Do you choose foods according to your mood? For example, when you are sad or irritated. | Not at all | Usually not | It depends | Mostly | Always |
| Q46 Do you take along healthy snacks when you go out? For example, nuts, vegetables, fruits, etc.. | Not at all | Usually not | It depends | Mostly | Always |
| Q47 Imagine that you are at a place where you see and smell tasty foods. Are you able to resist the temptation of buying them? For example, at train stations, department store food floors, bakeries, etc.. | Not at all | Not really | It depends | Somewhat | Yes |
| Q48 Are you able to say “no” to tasty sweets when you are tempted to have some? | Not at all | Not really | It depends | Somewhat | Yes |
| Q49 Are you able to eat healthily when the situation deviates from usual? For example, when you have unexpected guests, time constraints, etc.. | Not at all | Not really | It depends | Somewhat | Yes |
| Q50 Are you able to eat healthily when you feel stressed? | Not at all | Not really | It depends | Somewhat | Yes |
| Q51 We ask about food products that you keep at home. Do you keep healthy snacks for yourself at home? For example, nuts, vegetables, fruits, etc.. | Not at all | Hardly ever | It depends | Mostly | Always |
| Q52 Do you keep 4 or more packages of potato chips, rice crackers, or salty snacks at home? | Not at all | Hardly ever | It depends | Mostly | Always |
| Q53 Do you keep 4 or more packs of candy, cookies, or chocolate at home? | Not at all | Hardly ever | It depends | Mostly | Always |
| Q54 Do you keep 4 or more bottles of sugar-sweetened beverages (including sweetened tea, sports drinks, and juice with 30% fruit content) at home? | Not at all | Hardly ever | It depends | Mostly | Always |
| Q55 Do you keep 4 or more cartons of 100% fruit juice at home? | Not at all | Hardly ever | It depends | Mostly | Always |
| Q56 Do you purchase healthy foods even when you have limited money? For  example, vegetables, fruits, whole grain products, etc.. | Not at all | Usually not | It depends | Mostly | Always |
| Q57 Do you purchase healthy foods, even if they are a bit more expensive? For example, vegetables, fruits, whole grain products, etc.. | Not at all | Usually not | It depends | Mostly | Always |
| Q58 Do you check the nutritional labels of products for calories, fat, sugar, or salt content? | Not at all | Usually not | It depends | Mostly | Always |
| Q59 Do you compare the calories, fat, sugar, or salt content of different products? | Not at all | Usually not | It depends | Mostly | Always |
| Q60 Are you able to see, smell, or feel the quality of fresh foods? For example, meat, fish, fruits, etc.. | Not at all | Not really | It depends | Somewhat | Yes |
| Q61 We ask about your cooking skills. Are you able to prepare a meal using fresh ingredients (such as raw vegetables, meat, fish, etc.) without using pre-packaged and processed foods? | Not at all | Not really | It depends | Somewhat | Yes |
| Q62 Do you find it difficult to prepare a meal with six or more types of fresh ingredients? | Not at all | Not really | It depends | Somewhat | Very much |
| Q63 Are you able to prepare fresh fish in different ways? For example, simmering, steaming, stir-frying, etc., or using it in various dishes. | Not at all | Not really | It depends | Somewhat | Yes |
| Q64 Are you able to prepare fresh vegetables in different ways? For example, simmering, steaming, stir-frying, etc., or using them in various dishes? | Not at all | Not really | It depends | Somewhat | Yes |
| Q65 Are you able to make a recipe into your own version yourself? For example, if you are missing one of the ingredients, etc.. | Not at all | Not really | It depends | Somewhat | Yes |
| Q66 We ask about eating with others. Do you find it important to eat at the dinner table when dining with others? | Not at all | Not really | It depends | Somewhat | Very much |
| Q67 When you are with others, do you find it important to eat together? | Not at all | Not really | It depends | Somewhat | Very much |
| We ask about when and what you eat and drink. |  |  |  |  |  |
| Q68 Do you engage in any other activities while eating? For example, reading books, working, using your smartphone, watching TV, etc.. | Not at all | Usually not | It depends | Mostly | Always |
| Q69 Do you eat the total contents of a bag or container of potato chips, candy, cookies, etc. in one go? | Not at all | Usually not | It depends | Mostly | Always |
| Q70 Do you eat fruit as a snack? | Not at all | Usually not | It depends | Mostly | Always |
| Q71 Do you eat vegetables as snacks? | Not at all | Usually not | It depends | Mostly | Always |

**Questions regarding the child's mother (if you do not wish to answer, please select "I do not wish to answer."**

７２. How old is the child's mother?

| ① Teens | ② 20s | ③ 30s | ④ 40s | ⑤ 50s | ⑥ 60 years and older | ⑦ Do not want to answer |
| --- | --- | --- | --- | --- | --- | --- |

７３. What is the highest level of education of the child’s mother?

| ① High school | ② Vocational school | ③ Junior college | ④ University or higher | ⑥ Do not want to answer |
| --- | --- | --- | --- | --- |

７４. What is the current employment status of the child’s mother?

| ① Full-time housewife | ② Part-time/casual | ③ Regular employee | ④ Do not want to answer |
| --- | --- | --- | --- |

７５. Does the child’s mother drink alcohol?

| Never | Past | Current |
| --- | --- | --- |

７６. Does the child’s mother smoke cigarettes?

| Never | Past | Current |
| --- | --- | --- |

７７. With whom does the child’s mother live? (multiple answers allowed)

| Living alone | Spouse/Partner | Children | Father |
| --- | --- | --- | --- |
| Mother | Father-in-law | Mother-in-law | Prefer not to answer |

７８. Questions regarding the child’s mother's feelings about life. For each of the following, (A) to (C), please choose the number that best describes the mother's feelings, with ① being very applicable and ⑦ being not applicable at all.

(A) I believe I can find solutions to everyday difficulties and problems.

| ① Very applicable | ② | ③ | ④ | ⑤ | ⑥ | ⑦ Not applicable at all |
| --- | --- | --- | --- | --- | --- | --- |

(B) I believe that some of the difficulties and problems that arise in life are worth facing and working through.

| ① Very applicable | ② | ③ | ④ | ⑤ | ⑥ | ⑦ Not applicable at all |
| --- | --- | --- | --- | --- | --- | --- |

(C) I believe I can understand and anticipate the difficulties and problems that arise in my daily life.

| ① Very applicable | ② | ③ | ④ | ⑤ | ⑥ | ⑦ Not applicable at all |
| --- | --- | --- | --- | --- | --- | --- |

**This concludes the survey. Thank you for your cooperation.**
